# Spatially-Distributed Cost–Effectiveness Analysis Framework to Control Phosphorus from Agricultural Diffuse Pollution

**DOI:** 10.1371/journal.pone.0130607

**Published:** 2015-08-27

**Authors:** Runzhe Geng, Xiaoyan Wang, Andrew N. Sharpley, Fande Meng

**Affiliations:** 1 College of Resources, Environment and Tourism, Capital Normal University, Beijing, 100048, China; 2 Policy Research Center for Environmental and Economy, Ministry of Environmental Protection, P. R. China, Beijing, 100029, China; 3 Department of Crop, Soil and Environmental Sciences, University of Arkansas, Fayetteville, 72701, United States of America; NERC Centre for Ecology & Hydrology, UNITED KINGDOM

## Abstract

Best management practices (BMPs) for agricultural diffuse pollution control are implemented at the field or small-watershed scale. However, the benefits of BMP implementation on receiving water quality at multiple spatial is an ongoing challenge. In this paper, we introduce an integrated approach that combines risk assessment (i.e., Phosphorus (P) index), model simulation techniques (Hydrological Simulation Program–FORTRAN), and a BMP placement tool at various scales to identify the optimal location for implementing multiple BMPs and estimate BMP effectiveness after implementation. A statistically significant decrease in nutrient discharge from watersheds is proposed to evaluate the effectiveness of BMPs, strategically targeted within watersheds. Specifically, we estimate two types of cost-effectiveness curves (total pollution reduction and proportion of watersheds improved) for four allocation approaches. Selection of a ‘‘best approach” depends on the relative importance of the two types of effectiveness, which involves a value judgment based on the random/aggregated degree of BMP distribution among and within sub-watersheds. A statistical optimization framework is developed and evaluated in Chaohe River Watershed located in the northern mountain area of Beijing. Results show that BMP implementation significantly (*p* >0.001) decrease P loss from the watershed. Remedial strategies where BMPs were targeted to areas of high risk of P loss, deceased P loads compared with strategies where BMPs were randomly located across watersheds. Sensitivity analysis indicated that aggregated BMP placement in particular watershed is the most cost-effective scenario to decrease P loss. The optimization approach outlined in this paper is a spatially hierarchical method for targeting nonpoint source controls across a range of scales from field to farm, to watersheds, to regions. Further, model estimates showed targeting at multiple scales is necessary to optimize program efficiency. The integrated model approach described that selects and places BMPs at varying levels of implementation, provides a new theoretical basis and technical guidance for diffuse pollution management in agricultural watersheds.

## Introduction

Best management practices (BMPs) have been widely implemented in watersheds to trap and control P sources and transport from agricultural landscapes [1–4]. Many studies have clearly demonstrated the need for targeted BMP selection within a watershed (Chang et al., 2009; Qi et al., 2011; Shen et al., 2013). Thus, BMP optimization has become a critical component of effective non-point source pollution control strategies [5].

Recent studies have shown that the accuracy of BMP selection and placement can be enhanced by constructing a cost-effective decision support system that combines a process-based watershed model, an optimization algorithm, and an economic assessment tool [6–11].

The above optimization technology and theory have been efficiently utilized to quantify BMP effectiveness at field and watershed scales. However, practical applications have been limited for several reasons. First, spatial allocation BMPs were randomly distributed. Further, considering resource constraints, it is not possible to implement BMPs on every field in a watershed. Second, BMP placement on every field is not usually appropriate because small areas of a watershed often contribute disproportionately large amounts of pollutant loads. When selected for implementation in these critical zones, BMPs will achieve maximum reduction efficiency [3, 12, 13]. Third, nonpoint source models commonly allocate BMPs on a single spatial scale (field, farm or watershed), with evaluation indicators usually the mitigation efficiency of pollution load. Fourth, most optimization schemes are time consuming and computationally inefficient, given the potentially infinite number of BMPs placement scenarios in a watershed. The computation time for the optimization process was typically on the order of days) [14–17].

Considering the complexity and lack of field verification, considerable uncertainties exist because of difficulties in linking hydrologic response units (HRUs) with critical source areas (CSAs) and BMP effectiveness through individual hydrology models [18]. Moreover, natural farm boundaries seldom coincide with HRU boundaries in hydrology models. Watershed-scale models based on hydrologic boundaries (i.e., SWAT, AnnAGNPS, and Hydrological Simulation Program—Fortran or HSPF models) are employed to identify and estimate CSAs and the impact of BMPs on water quality. However, BMPs are selected, implemented, and maintained at the farm- or field-scale and applied within the field and farm boundaries [8].

BMP effectiveness is generally determined as a percent difference in P loss before and after implementing BMPs [19]. However, the improvements in water quality in response to BMP intervention are not always reflected in watershed-scale nutrient flux reductions [20, 21]. This may be due to;

1. the hydrologic model overestimates BMP effectiveness [22],
2. the time lag effect between BMP effectiveness and significant changes in water quality [23], and
3. although the reductions of BMP would have occurred, but it cannot detectable due to the background variability in local pollutant loads driven by the uncontrolled weather or natural factors [24].

Much of the recent literature has focused on the concept of complex, adaptive cost-effectiveness analysis systems for BMP placement at a single spatial scale, with limited approaches to address adequately the complexities of these systems at multi-spatial scale watershed.

The watershed-scale model, HSPF (Hydrological Simulation Program-Fortran), was selected in this study for identifying and quantifying P loss at a subwatershed-level. HSPF simulates for extended periods of time the hydrologic, and associated water quality, processes on pervious and impervious land surfaces and in streams and well-mixed impoundments. It has been applied extensively around the world [25–28]. The GIS interface with which the model is integrated aids spatially mapping model-predicted CSAs of P loss.

Risk assessment tools can identify key nutrient source and transport processes at the field scale [19]. The P Index take into account source and transport factors to describe nutrient availability, and erosion, runoff, leaching and connectivity to account for delivery. In this study, an integrated approach was developed, it includes: field runoff monitoring, risk assessment model simulation techniques for assessment and identify the CSAs areas, BMP assessment tool for calculate the effectiveness of BMPs, and statistical analysis to identify the optimal location for BMPs and to estimate trade-offs at various scales before and after implementation.

The objectives are as follows: (1) identify the spatial distribution frequency of P pollution loads at two scales (subwatershed and field); (2) examine the relationship between the probability of statistically significant water quality improvement and reduction proportion of pollution load in a watershed and generate effect evaluation indices; and (3) distinguish the change trends and benefit/cost curves of four approaches to geographically allocate conservation efforts. This work is helpful in further controlling soil erosion and non-point source pollution and containing the Miyun Reservoir, China.

## Study Area

**Fig 1:**
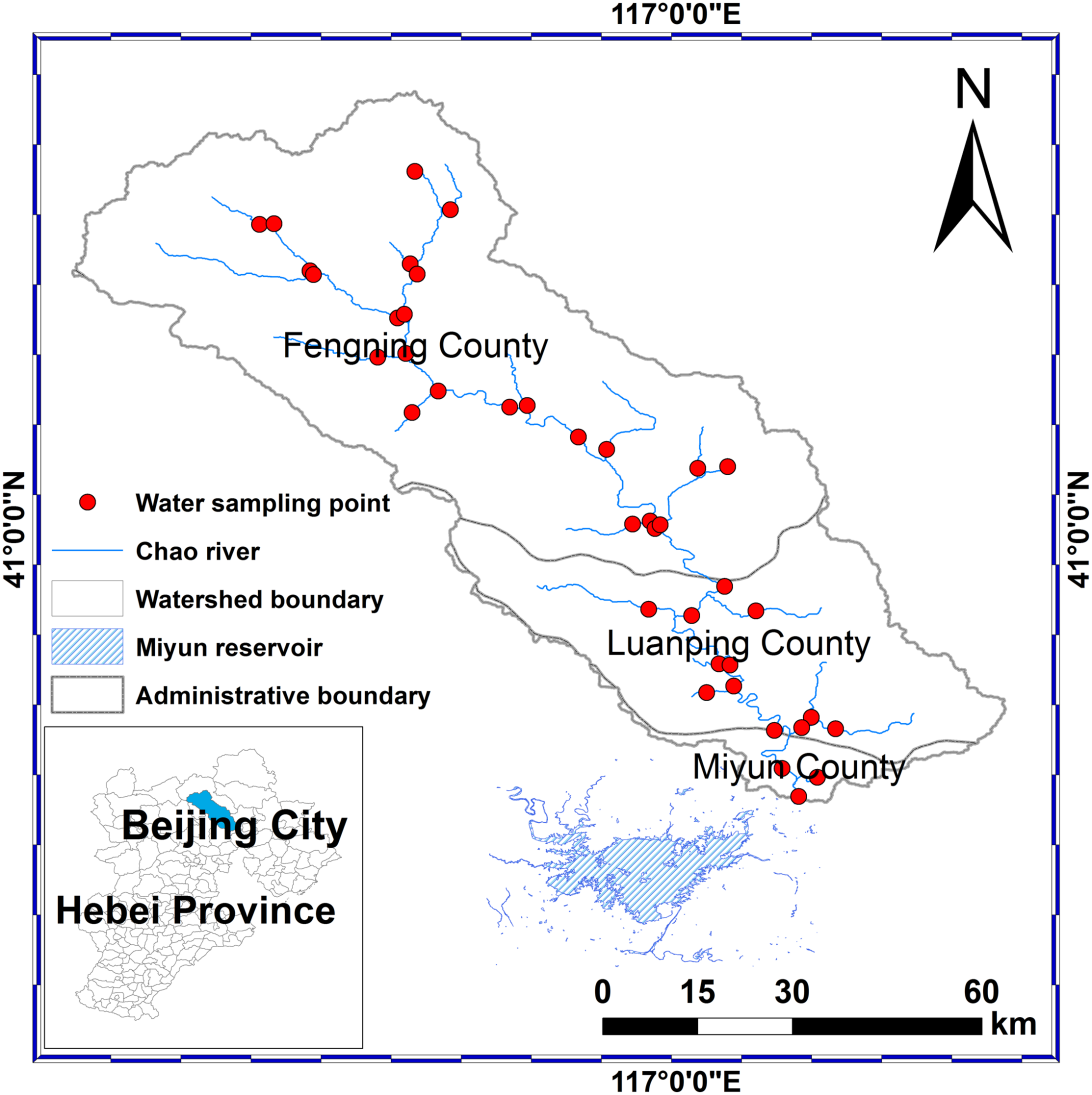
Location of Chaohe River watershed in Beijing and Hebei province, location of China.

Chaohe River Watershed is located in North China; it has a drainage area of 4888 km^2^ containing Miyun Reservoir, Beijing’s drinking water supply (Fig 1). Around the watershed, there is no large city, only a few small factories in Hebei Province, and the economy is dominated by agriculture. Nearly all agricultural activities and residents locate close to the banks of Chaohe River. Therefore, Miyun Reservoir continues to face a serious threat of eutrophication from non-point sources following point source controls in Miyun Reservoir Watershed [29, 30]. The study area experiences a temperate, semi-arid, sub-humid continental monsoon climate with an annual mean precipitation of 600 mm, 77% of which falls in July to September. The soils of Chaohe River Watershed are typically Argosols, Cambosols and Aridosols, namely Luvisols, Alisols, Cambisols, Calcisols and Gypsisols, based on World Reference Base for Soil Resources. Land use types include farm land (8%), forest land (51%), grassland (34%), water (4%), urban land (2%), and unused land (1%).

## Methodology

**Fig 2:**
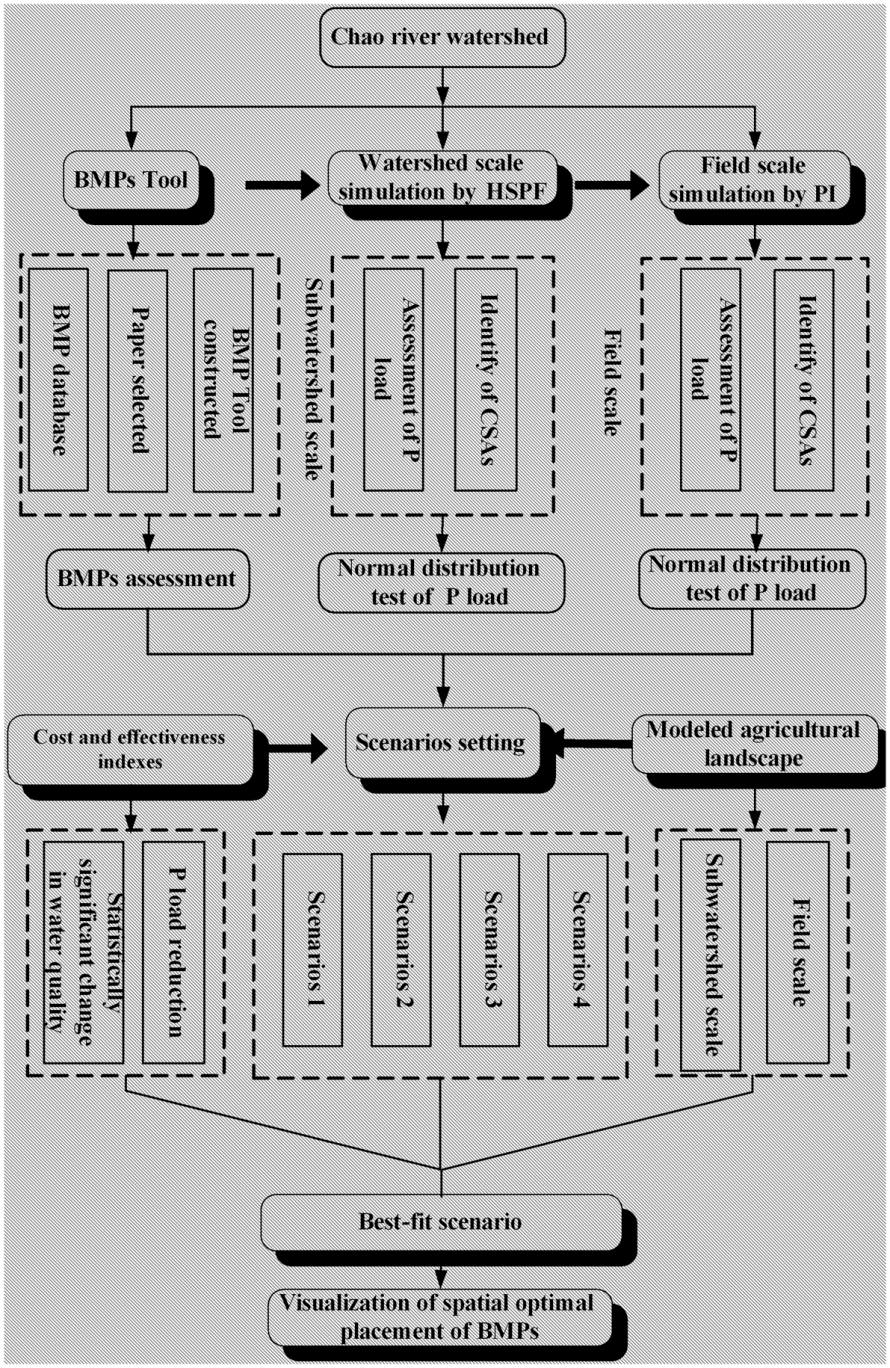
Flow chart of integrating modeling framework.

The modeling framework (Fig 2) represents a multi-scale approach to the multi-objective task of mitigating P pollution and statistically improvement the water quality of whole watershed.

### 3.1 Data preparation

The model input data utilized were a combination of the following:

1. A digital elevation model (DEM) constructed by the Institute of Geographical and Natural Resources Research, CAS (Chinese Academy of Sciences), that provides a consistent coverage of topography at a resolution of 30 m;
2. A land use map with a scale of 1:100,000 constructed by digitalizing and interpreting remote sensing images provided by the Institute of Geographical and Natural Resources Research, CAS;
3. A soil type scale of 1:100,000 constructed by digitalizing and interpreting remote sensing images provided by the Institute of Geographical and Natural Resources Research, CAS;
4. Weather data (1991 to 2010) collected from 12 stations in the study area by China Meteorological Administration.

Hydrological (1991 to 2010) and water quality (1996 to 2010) data were obtained from relevant monitoring stations throughout the study area.

### 3.2 HSPF model simulation

Chao River Watershed was divided into 97 subwatersheds according to the drainage area and model calculations by HSPF model. The manual calibration procedure based on the trial and error process of parameter adjustments was used and simulations performed by changing the calibration parameters. After adjustment for each parameter, the simulated and measured runoff and total P (TP) yield were compared to judge the improvement in the model prediction. Some of the HSPF parameters governing the estimations of runoff, sediment and TP and used in the model calibration are given in Table 1. Their calibrated values for the watershed along with their possible range are also given in Table 1.

**Table 1:** Parameters Used in Runoff, Sediment and TP Yield Calibration of HSPF Model.

Process parameter	Description	Calibrated watershed parametric values	Possible range of value	
			Minimum	Maximum
Runoff calibration parameters				
LZSN	Lower zone nominal storage	5	5	7.5
INFILT	Index to the infiltration capacity of the soil	0.1	0.03	0.5
AGWRC	Base groundwater recession	0.98	0.001	0.999
DEEPFR	Fraction of GW inflow to deep recharge	0.1	0.0	1.0
UZSN	Upper zone nominal soil moisture storage	1.128	0.25	0.75
INTFW	Interflow inflow parameter	7.5	1	7.5
IRC	Interflow recession parameter	0.5	0.3	0.85
Sediment yield calibration parameters				
KRER	Coefficient in the soil detachment equation	0.2	0.15	0.4
JRER	Exponent in the soil detachment equation	2.0	1.0	3.0
KSER	Coefficient in the detached sediment washoff equation	6	0	10
JSER	Exponent in the detached sediment washoff equation	1.9	1.0	3.0
TP calibration parameters				
KDSP	first order phosphate desorption rate	0.03	0	0.5
KADP	first order phosphate adsorption rate	0.07	0	1.0
KIMP	first order phosphate immobilization rate	0.025	0	5.0
KMP	first order organic P mineralization rate	0.05	0.0001	0.03

**Fig 3:**
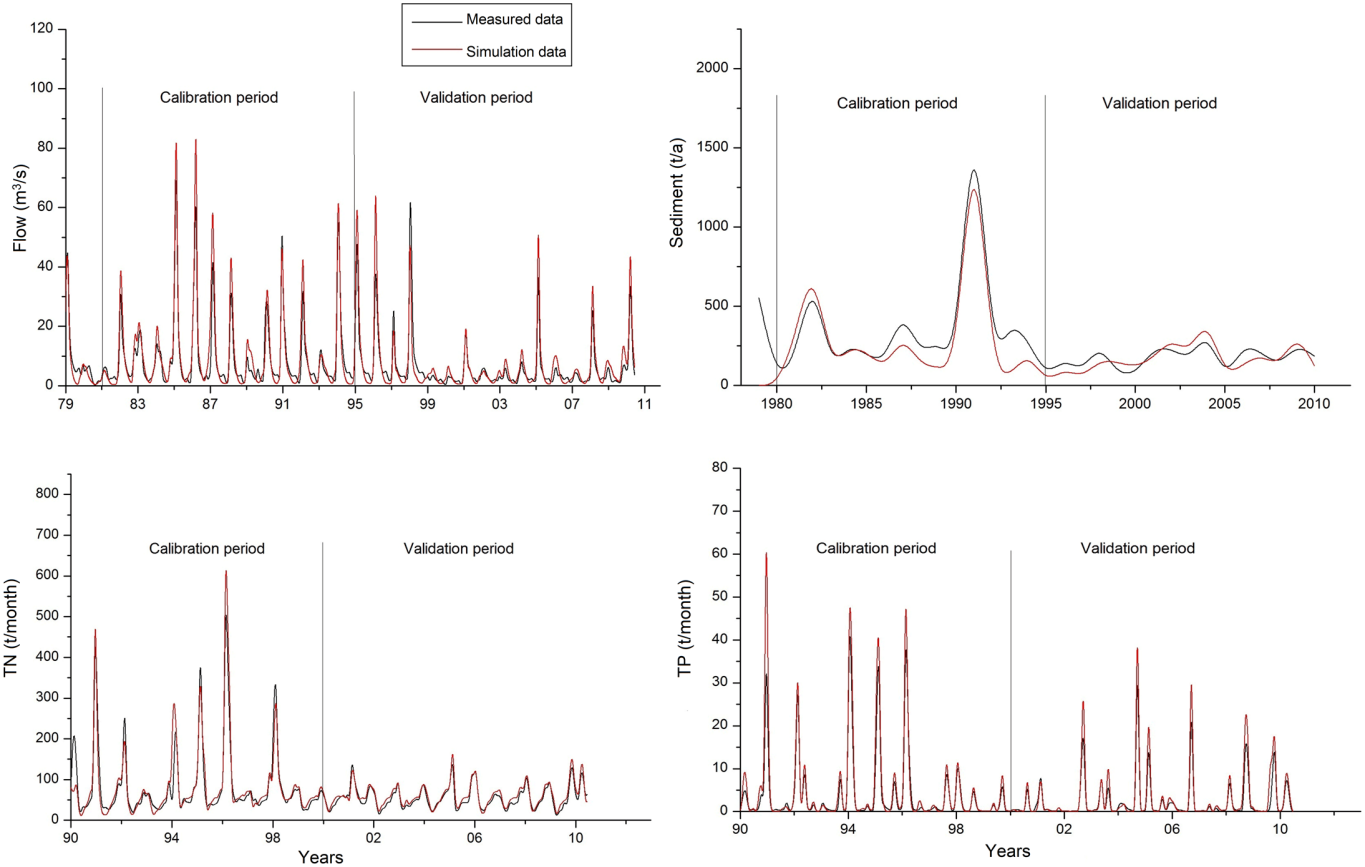
Calibration and validation of HSPF model.

Calibration and validation were conducted based on observed data by adjusting the key parameters until the simulation values were reasonably close to the observed values (Fig 3). First, flow calibration and validation were conducted. Observed flow data from 1990 to 2000 were utilized to calibrate the parameters with the total relative error of flow simulation at different intervals of the year, season, month, and single storm event. Verification of data from 2001 to 2010 was then conducted. Second, sediment yield was calibrated and validated. Lastly, total phosphorus (TP) yield was calibrated and validated. The procedure was performed similar to the hydrological calibration and validation process. The accuracy of flow simulation is the precondition of accuracy in sediment and nutrient simulation. To evaluate the model’s goodness of fit, the most commonly used statistical measures for model assessment, Nash efficiency coefficient (*E*_*ns*_) and relative error (*RE*), were employed [31].

The results of *E*_*ns*_ and *RE* indicate that the output estimates from the HSPF model can serve as satisfactory and acceptable datasets for the further analysis of BMP selection and placement (Table 2).

**Table 2:** Nash efficiency coefficient (Ens) and relative error (RE) of HSPF simulation.

	Nash efficiency coefficient (Ens)		Relative error (RE)	
	Calibration	Validation	Calibration	Validation
Flow	0.88	0.89	14.50%	13.10%
Sediment	0.82	0.79	15.36%	16.67%
TP		0.63		40.72%

### 3.3 Risk assessment with the P index

A modified P Index was developed to represent field-scale P inputs and outputs of non-point source pollution by analyzing local hydrological and meteorological data, land use, soil, soil conservation, farmland management, population density, and livestock. The factors of livestock and population density are new factors added to the P Index system based on the actual local characteristics [30]. The final index was adjusted to a suitable size (30 m) of CSAs to assign sources and target-oriented control measures based on spatial and statistical analyses conducted with the ArcGIS 10.1 platform. Unlike most current PI that allow for minimal calibration to estimate the risk of P loss (dimensionless values)[13], the PI of Chaohe River Watershed estimates actual loss according to the quantitative relationship between P loss risk estimates at the field scale and P loads from the relevant watershed; the index has been validated through annual runoff monitoring in 10 standard plots in Shixia Watershed downstream of Chaohe River Watershed [32].

### 3.4 Effectiveness of BMPs

A means of estimating BMP effectiveness was constructed based on data from almost 300 studies on BMP effectiveness published in China and the United States [33]. A tool (database) that allows users to determine BMP effectiveness according to soil type and slope conditions at a given site was developed. This database includes 60 agricultural BMPs grouped into six classes (S1 File). The database was assessed through analysis of variance to establish the effects of primary factors believed to influence BMP effectiveness. Data from combined soil and slope analyses were utilized to design a BMP effectiveness estimator based on user-specified hydrologic soil groups and slope classes. Visual Basic for Applications and structured query language were employed to design the said estimator. The BMP database provides an estimate of the costs and effectiveness of each BMP that can be implemented at a specific field scale in the watershed [33].

### 3.5 BMP spatial distribution scenarios

Four BMP implementation scenarios were considered under the following principles [34].

**Fig 4:**
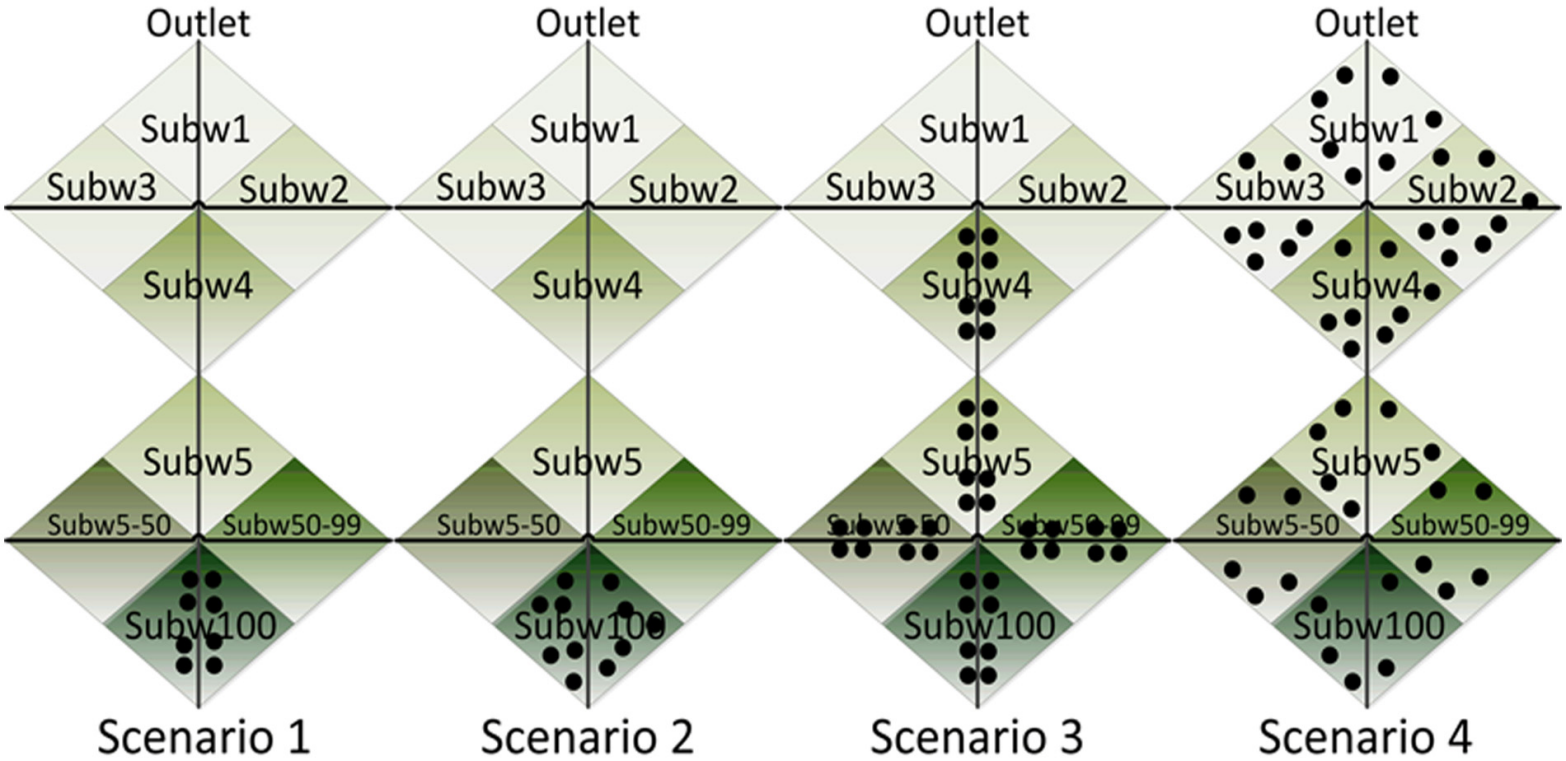
Four sets of BMPs are located in the conceptual watershed. Note: Black lines represent a stream network; spatially-independent subwatersheds are shaded ascending order (darker = higher) based on their proportion of P loads account for whole watershed P loads; Black dots are targeted fields for BMPs implemented, illustrative purposes, locations near streams means reduce more pollution).

1. Targeted placement of BMPs is applied in certain subwatersheds with relatively high P loads (aggregated).
2. BMPs are randomly distributed without regard for watershed boundaries and P loads (dispersed).
3. BMPs are targeted to a percentage of field P load in the entire watershed without regard for watershed members (dispersed).
4. BMPs are randomly implemented on fields in the subwatershed with the highest P loads. The same procedure is then applied to the subwatershed with the second highest P load and so on (aggregated) (Fig 4).

Synthetic data where come from the PI, HSPF and BMP tool and a conceptual case study can clarify the analysis results and reduce the problems resulting from the limitation of measured data. A model agricultural landscape was developed to compare various approaches for allocating conservation efforts at the watershed scale [35, 36]. If proportional P loss distributions within the watershed are similar in shape and log-normal, then the distribution of individual field P losses is also log-normal.

In the present study, the construction of a conceptual watershed was based on methods developed by Hsieh et al. (2007) and Diebel et al. (2008) [35, 37]. We compared four scenarios of allocating BMPs in a conceptual watershed; the effectiveness of these scenarios was limited to reduce P loss. Each scenario is composed of 100 equally sized and spatially independent model subwatersheds and 10,000 model fields randomly selected from the Chaohe River Watershed. The subwatershed loads were converted to proportions of the entire watershed load. Similarly, P loads from fields were converted to subwatershed loads in the form of proportions for a certain subwatershed. Thus, the standardized entire watershed P load (*λw*) was set to 1. A detailed derivation process can be found in the paper written by [35]. The proportional contribution of the field to the entire watershed P load can be expressed as

$$\lambda_{w}=\overset{100}{\underset{subw=1}{\sum }}\lambda_{subw}=\overset{10000}{\underset{f=1}{\sum }}\lambda_{f,subw}=1,$$ (1)

where *λw* is the P load from the entire watershed, *λ*_*f*,*subw*_ is the proportion of each subwatershed P load in the watershed load, and *λ*_*f*_ is the proportion of watershed P load from each field.

### 3.6 Cost and effectiveness indexes

An index that describes the cost and benefits (effectiveness) of the implemented BMPs was established. The index can effectively represent the change in water quality for pollution reduction. Two types of cost—effectiveness curves (P load reduction and proportion in the entire water that achieved statistically significant water quality improvement) were estimated for the four BMP scenarios. The first type is the proportion of P loads in the whole watershed after BMPs were implemented. The second is the proportion of subwatersheds where a statistically significant reduction in the stream water P concentration is observed (measured water quality change) [38–40]. A ‘‘best-fit scenario” was selected based on the relative importance of the two types of effectiveness. Finally, a sensitivity analysis was conducted to identify and test the stability of the BMP scenarios under the influence of various model input uncertainties.

### 3.6.1 Relationship between statistically significant change in water quality and P load reduction

The Kruskal—Wallis test was applied to test *P*_*subw*_, which is the probability of achieving statistically significant changes in water quality at the outlet of a given subwatershed based on the BMP implementation level in that watershed. The test was implemented with SPSS 18.0 (BMP implementation level is represented by *I*_*subw*_, which is defined as the proportion of 100 fields in each watershed where BMPs are implemented). Subsequently, the *P*_*subw*_ for the four BMP allocations was estimated.

We selected a dataset that consists of time series (monthly samples from May to October in 2006 to 2010) of P concentrations from 30 outlets of the subwatershed of Chaohe River Watershed. The dataset also provides the best available estimate of P variability in Chaohe River streams. Log-normal frequency distributions were fittedto the sample values for each stream and then 24 random numbers from each distribution were generated to obtain water quality data before BMP implementation [35]. We then multiplied each value by *R*_*subw*_ (ranges from 0 to 1, increments of 0.05) to obtain water quality data after BMP implementation. Therefore, the sample means and standard deviations can be proportionally scaled by this procedure, in accordance with the trend seen across the range of measured values (σ = 0.62μ- 0.012, intercept not different from zero, *r* ^2^ = 0.63). Based on these two datasets, we applied the Kruskal—Wallis test to compare the median of water quality before and after BMP implementation in each outlet at every *R*_*subw*_ level [*P*_*subw*_(*R*_*subw*_); *p*<0.01]. A statistically significant improvement in water quality at each *R*_*w*_ can then be estimated. Furthermore, we can fit the relationship between *P*_*w*_ and *R*_*w*_ to obtain the following regression.

$$p_{subw}=\frac{1.059}{1+60\left({\text{e}}^{-13.2\cdot R_{subw}}\right)}-0.059$$ (2)

From the field scale to the entire conceptual watershed, *P*_*subw*_(*R*_*subw*_) and *R*_*f*_ (effectiveness of the selected BMP in terms of P loss from individual fields) were utilized to calculate *P*_*subw*_ (*R*_*f*_, *I*_*subw*_) in the four BMP allocation scenarios. *R*_*subw*_ can be expressed as

$$R_{subw}=R_{f}(\overset{100\cdot I_{subW}}{\underset{f=1}{\sum }}\lambda_{f,subw}).$$ (3)

Fields were selected in the order of highest to lowest *λ*_*f*,*subw*_ in Scenarios 1 and 3 and in a random order in Scenarios 2 and 4.

### 3.6.2 Development of P load reduction benefits

Through the field selection approach defined above, the modeled pollutant reduction benefit index *R*_*w*_ (P load reduction in the entire watershed) was calculated as follows:

$$R_{w}=R_{f}\left(\overset{10,000\times I_{w}}{\underset{f=1}{\sum }}\lambda_{f,subw}\right)$$ (4)

The measured water quality change benefit index ${\bar{P}}_{subw}\left(R_{f},I_{subw}\right)$ was calculate as Eq (5), it equivalent to the proportion of subwatersheds where a significant P reduction is detected. In Eq (5), the *P*_*subw*_ (*R*_*f*_, *I*_*subw*_) was calculated separately for each subwatershed based on Eqs (2) and (3) defined in Section 2.4.1 [35]:

$${\bar{P}}_{subw}\left(R_{f},I_{subw}\right)=\frac{\overset{100}{\underset{subw=1}{\sum }}P_{subw}\left(R_{f},I_{subw}\right)}{100}$$ (5)

## Results

### 4.1 P load distribution at different spatial scales

**Fig 5:**
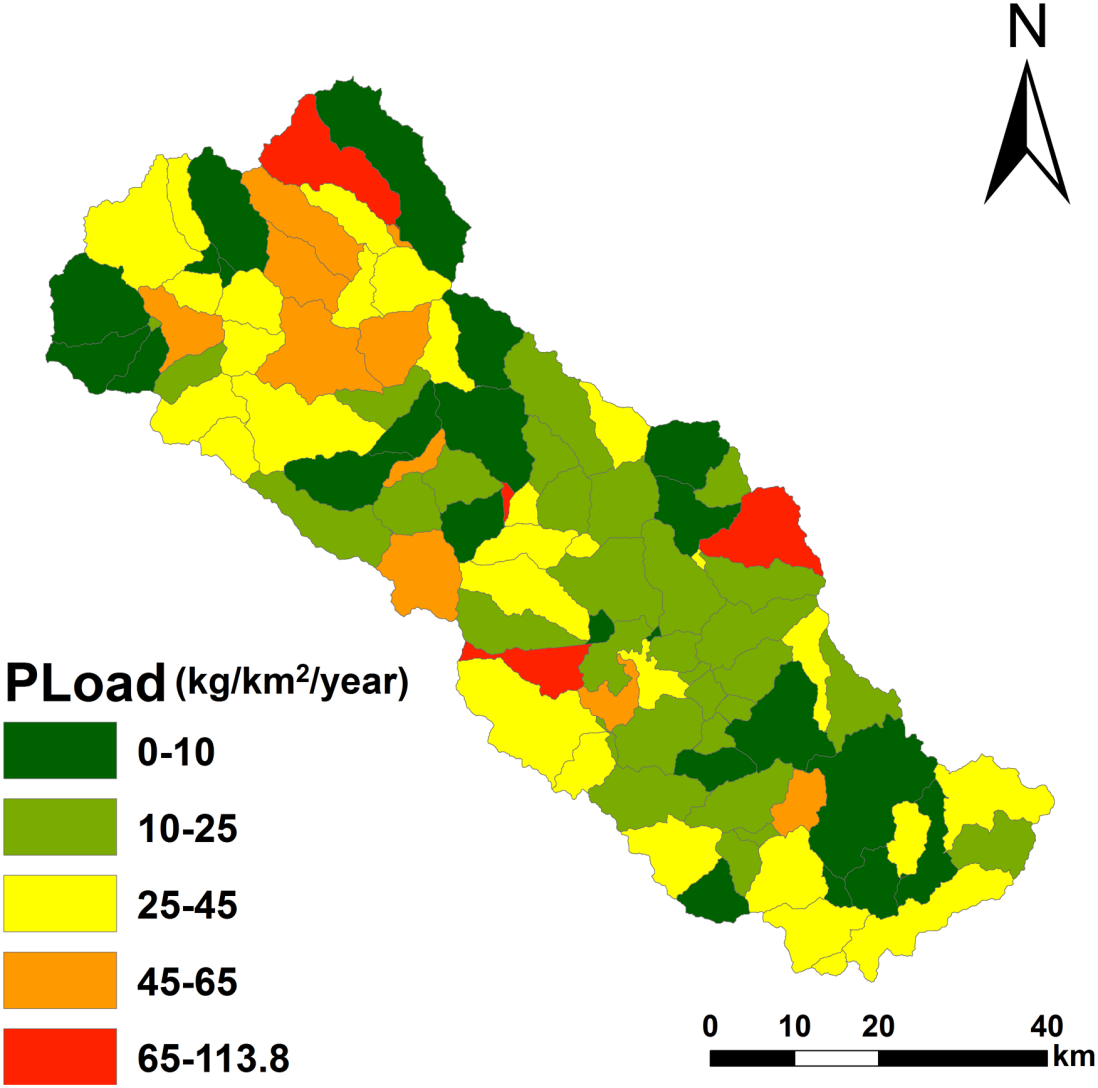
Spatial distribution of the P from HSPF model.

The P load from Chaohe River flows into Miyun reservoir mainly from July to September (flood season). The loss of sediments during flood season accounts for 78% to 90% of the value for the entire year. Thus, flood season is a critical period for soil erosion control and non-point source pollution prevention. For each subwatershed, the average pollution load is 21.32 kg, the maximum load is 113.85 kg, and the minimum value is 0.91 kg (Fig 5) (S2 File).

**Fig 6:**
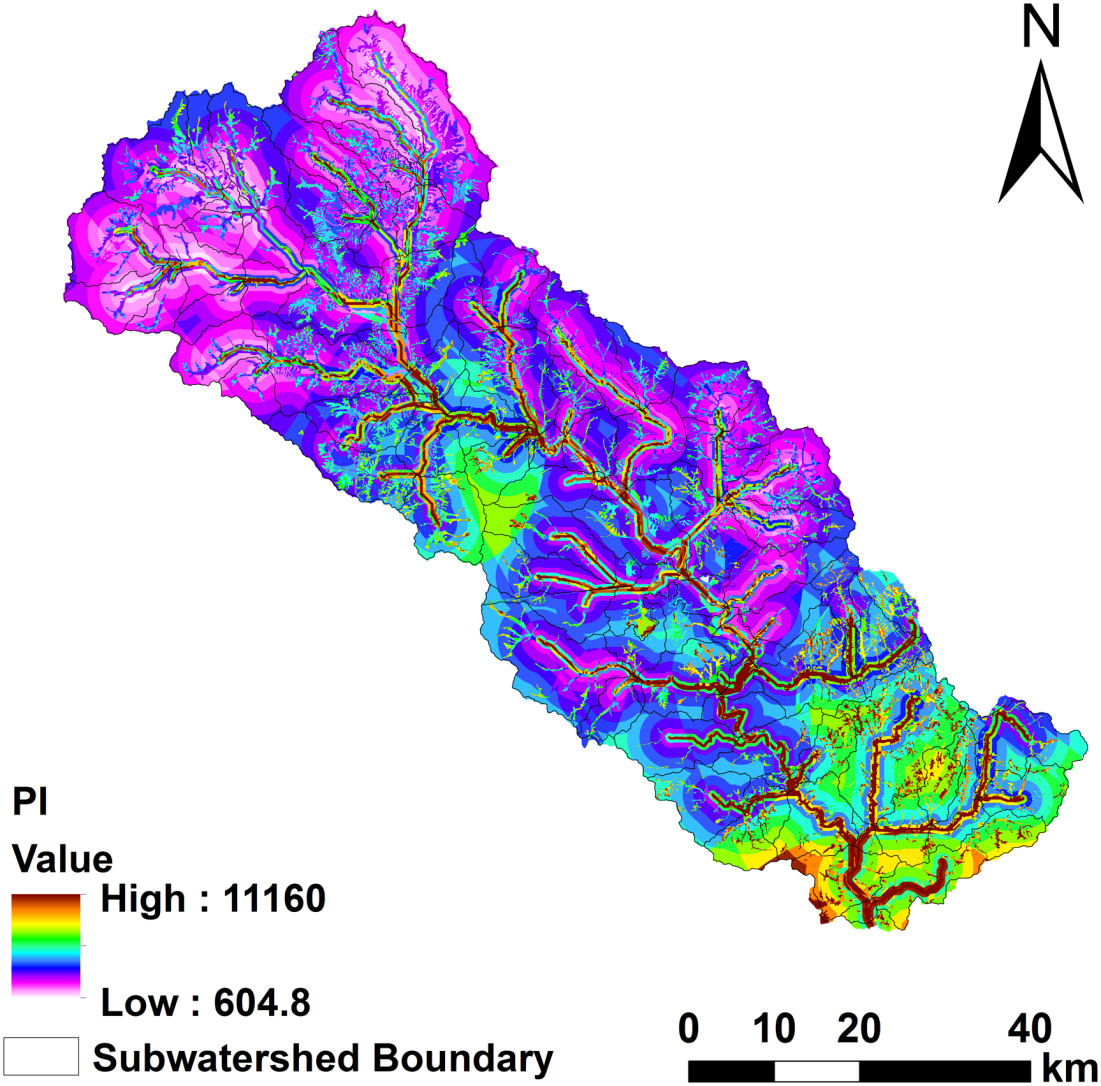
Spatial distribution of PI at field scale from PI model.

**Fig 7:**
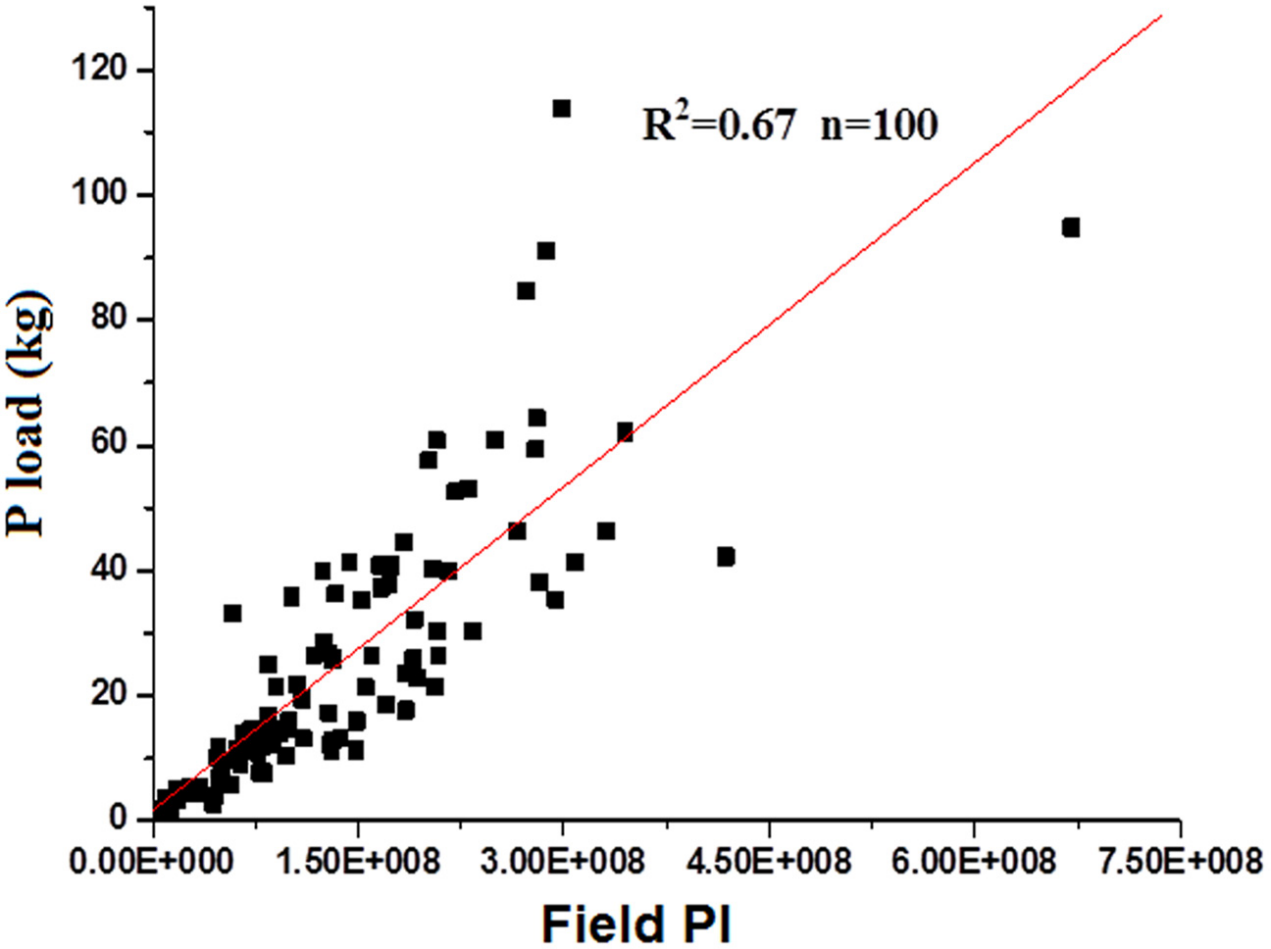
Correlation of watershed PI and the actual loss.

The PI results show that high-, moderate-, and low-risk areas account for 7.95%, 19.63%, and 72.42% of the total area, respectively (Fig 6) (S3 File). PI is significantly correlated (R^2^ = 0.67) with the actual loss at the watershed scale (Fig 7). The average load of P at the field scale is 2.94 kg, and the maximum and minimum values are 17.24 and 2.07 kg, respectively, exhibiting a spatial normal distribution.

**Fig 8:**
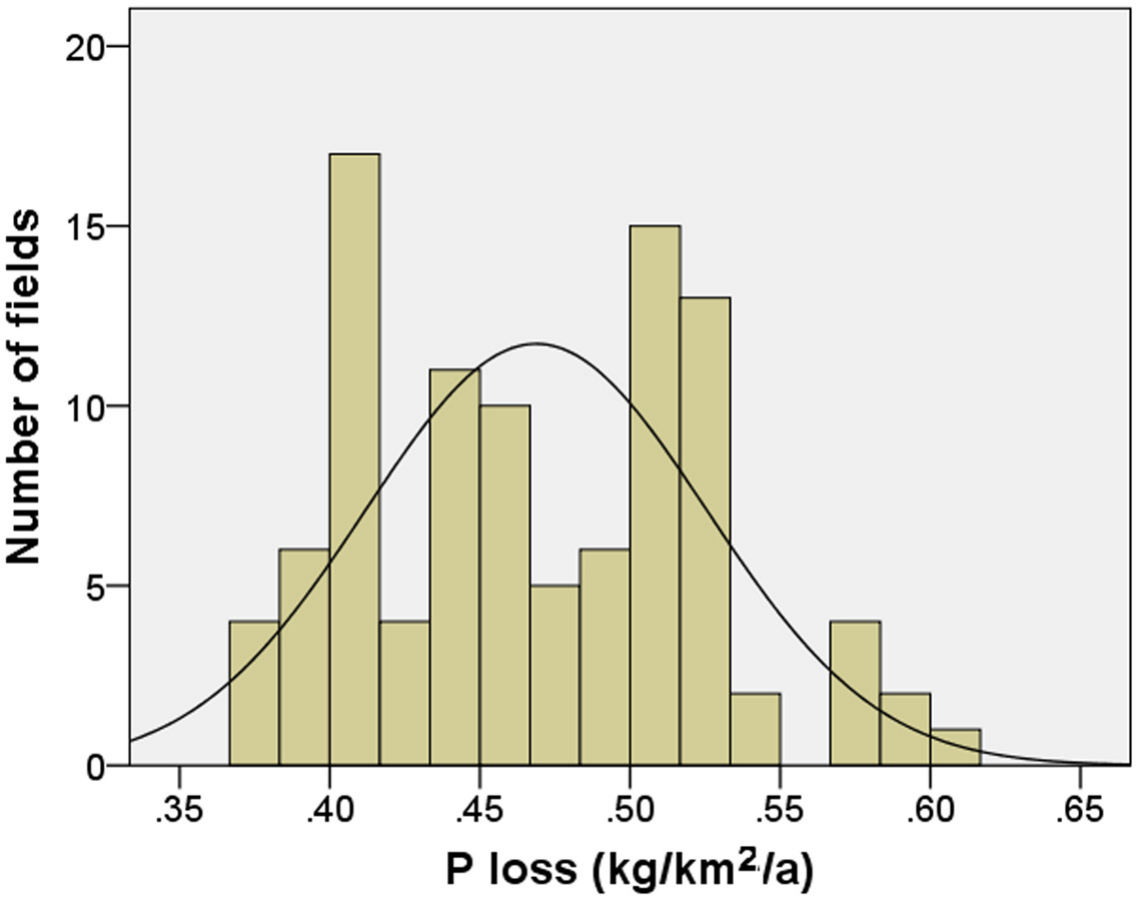
Log-normal distribution of phosphorus pollution at field scale.

**Fig 9:**
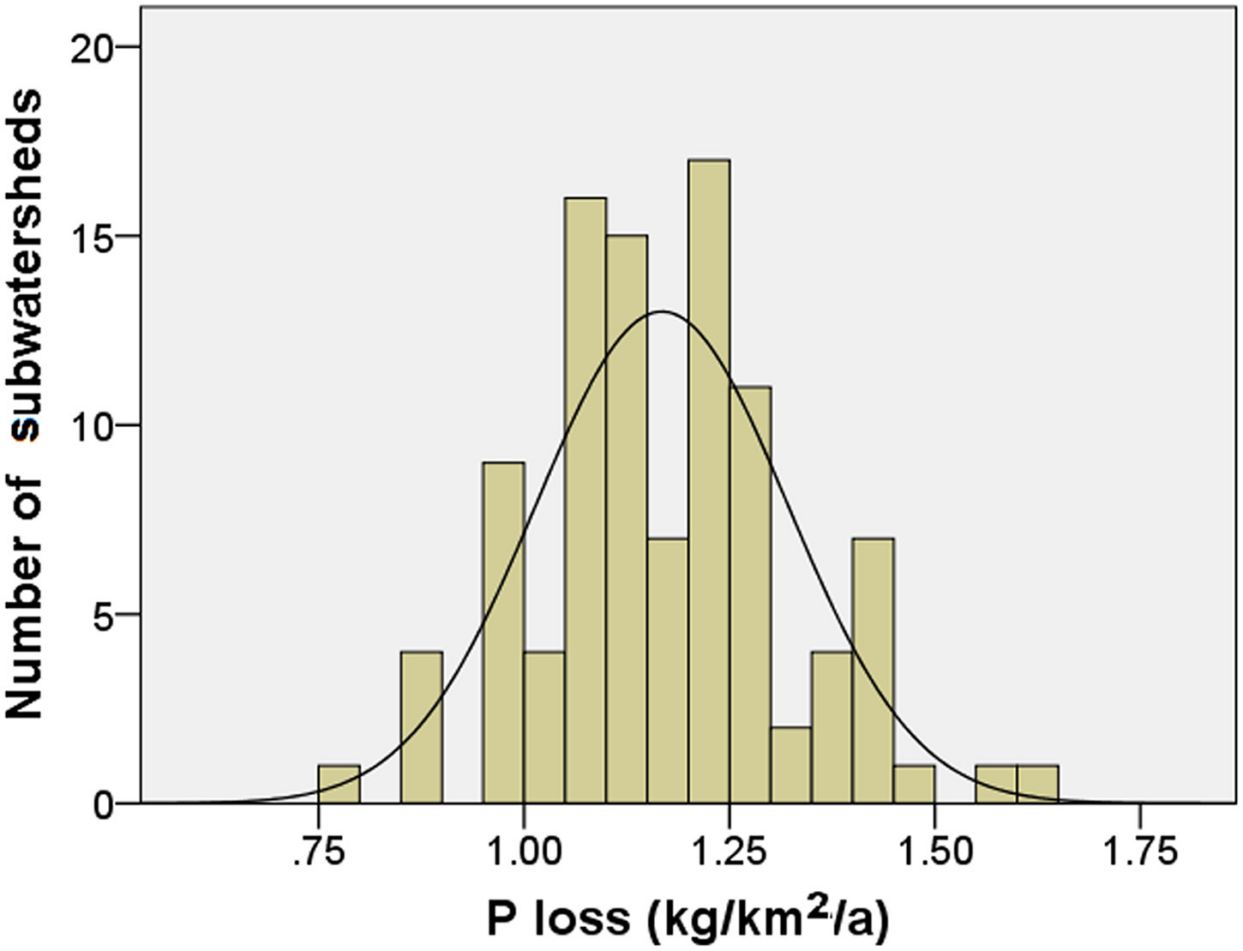
Log-normal distribution of phosphorus pollution at subwatershed scale.

We examined empirical P load data from HSPF and PI models through SPSS 18 to obtain evidence on the distribution situations at field at subwatershed spatial scales. At the field scale, we employed P loss values for 495 randomly selected agricultural fields with a mean area of 0.05 km^2^ in Chaohe River Watershed; the frequency distribution of P loss estimates in Chaohe River fits a log-normal distribution (Kolmogorov—Smirnov test: *P*_*normal*_ = 0.027 and *P*_*log-normal*_ = 0.198; S* = 0.05 and μ = 0.47) (Fig 8). At the subwatershed scale, annual unit area total P loads (kg/km^2^/year) were selected for 100 small watersheds (6.9 km^2^ to 18.3 km^2^) in Chaohe River Watershed with more than 40% agricultural land. The frequency distribution of P load estimates also fits a normal and log-normal probability distribution (Kolmogorov—Smirnov test: *P*_*normal*_ = 0.007 and *P*_*log-normal*_ = 0.334; S* = 0.15 and *μ* = 1.17) (Fig 9).

All of the frequency distributions analyzed demonstrate that the results from PI and HSPF models are ideal for the construction of a conceptual watershed for Chaohe River Watershed.

### 4.2 Prioritization and estimation of BMPs

A BMP tool was constructed to provide options quickly and simply based on reported effectiveness. In Chaohe River Watershed, Luvisols soil (HSG—B) that accounts for over 70% of land use and below 50% slopes was determined to heavily contribute to nutrient and sediment pollution according to the results of HSPF and PI simulations. We selected site properties similar to the observed values (HSG—B and slopes of 0% to 50%) and chose the best related BMP category for probable adoption in the watershed. In this case, nutrient control is implemented via structural methods and livestock/manure management to reduce P losses. Our tool provides effectiveness estimates of the applicable BMP classes for the specified site conditions and category. The tool provides results for two BMP classes that can be utilized in the watershed. Nutrient control by structural methods and livestock/manure management is potentially effective in reducing P losses. The average effectiveness values of nutrient control by structural methods and livestock/manure management for TP are 70% and 60%, respectively.

Based on reports on the effectiveness of BMPs implemented in Chaohe River Watershed, we assumed that BMP implementation would reduce P load from an individual field by 65% to simplify the calculations involving cost—effectiveness indexes and referred to the methods provided by [35, 41]. We also assumed that the cost of BMP implementation is constant among fields. Thus, the cost of BMP implementation serves as the BMP implementation level (number of fields or subwatersheds that implemented BMPs) and effort (size of BMPs for a given field).

### 4.3 Relationship between P load reduction and probability of obtaining a statistically significant P concentration

**Fig 10:**
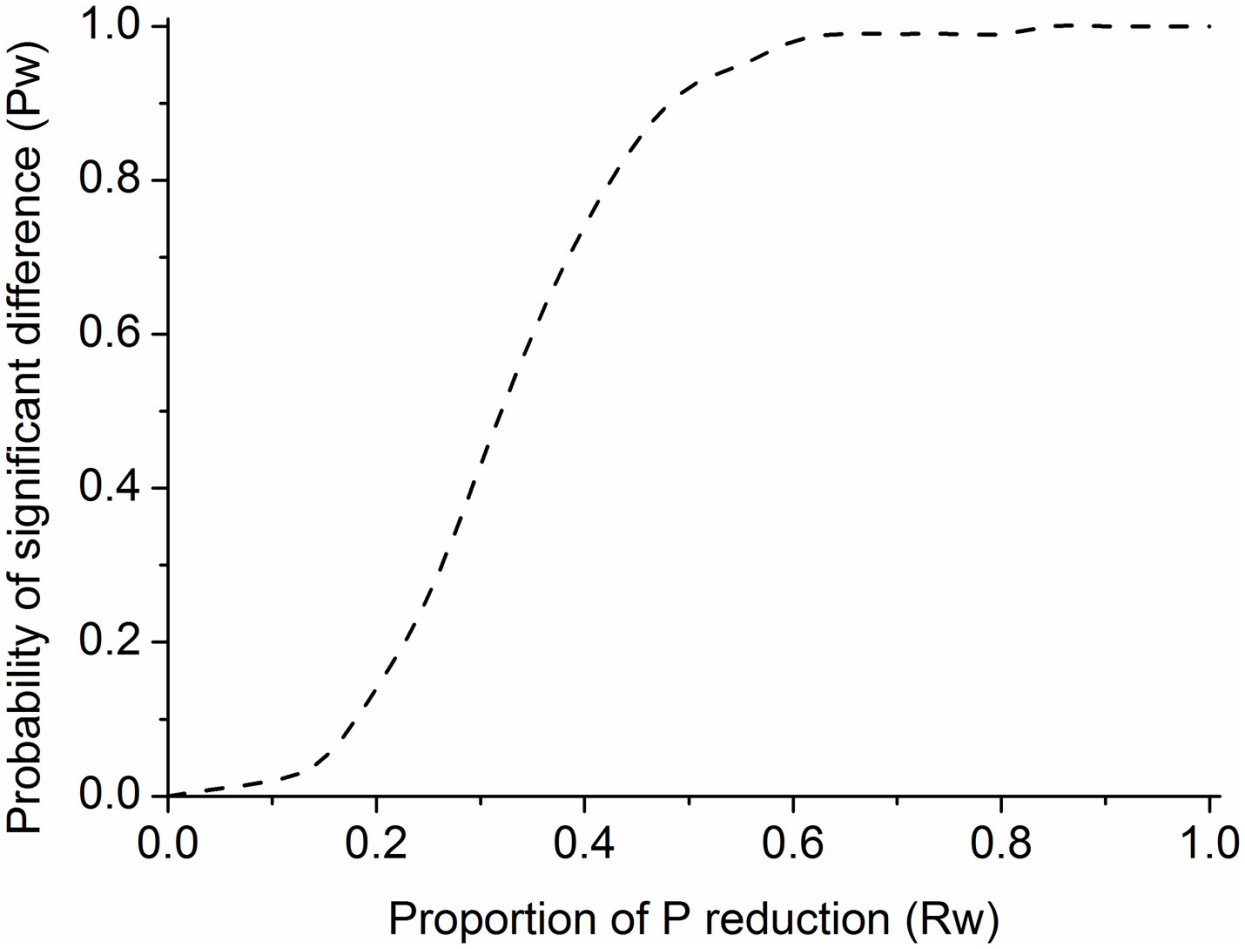
Test results from Kruskal Wallis method. Note: The probability (*P*_*subw*_) of detecting a statistically significant difference (P>0.01) in stream phosphorus concentration is plotted against the proportional phosphorus load reduction (BMP implemented level, *R*_*subw*_).

The probability of obtaining a statistically significant reduction in P load estimates after BMP implementation in Chaohe River Watershed is represented by a sigmoid (threshold) function curve (Fig 10) (S4 File) The probability of having a statistically significant improvement in P loss at each *R*_*w*_ level is shown in Table 3. The given streams show an abrupt threshold in the probability of water P concentration; a statistically significant improvement occurred when the proportion of P load reduction reached the 0.65 level in a certain subwatershed. As shown in Table 3, *R*_*subw*_ = 0.65 can serve as the threshold, where the targeted effectiveness of BMP is reached. A statistically significant improvement in water quality can be obtained in Chaohe River Watershed because as the effectiveness continues to increase, the cumulative probability density function for a randomly selected stream becomes more gradual.

**Table 3:** Dataset of Kruskal Wallis test.

*R*_*subw*_	0.05	0.1	0.15	0.2	0.25	0.3	0.35	0.4	0.45	0.5
*P*_*subw*_	0.00	0.00	0.05	0.14	0.26	0.43	0.60	0.74	0.85	0.92
*R*_*subw*_	0.55	0.6	0.65	0.7	0.75	0.8	0.85	0.9	0.95	1
*P*_*subw*_	0.95	0.98	0.99	0.99	1.00	1.00	1.00	1.00	1.00	1.00

### 4.4 Optimization of BMP allocation scenarios

**Fig 11:**
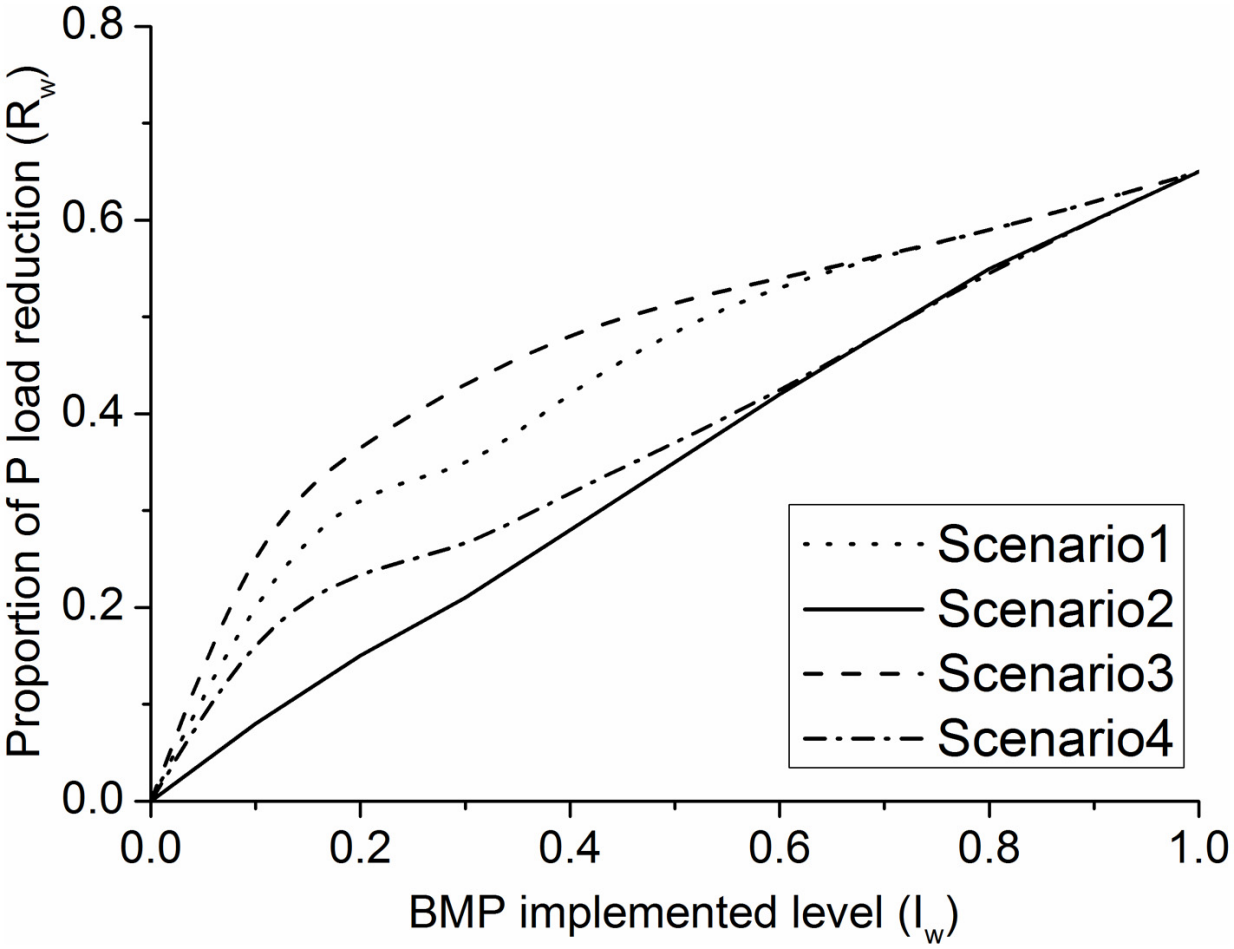
Cost-effectiveness curves for the *R*_*w*_ and *I*_*w*_ under four BMP scenarios.

We employed cumulative net benefit as a common estimate criterion to effectively present the two benefit indexes. In terms of the cumulative proportion of watershed P loads after BMP implementation (*R*_*w*_, first index) (Fig 11) (S5 File), Scenario 3 produced the best cost—effectiveness curve at all BMP implemented levels compared with the other three scenarios; Scenario 3 is followed by Scenarios 1, 4, and then 2, which produced the least benefit at all BMP effort levels.

**Fig 12:**
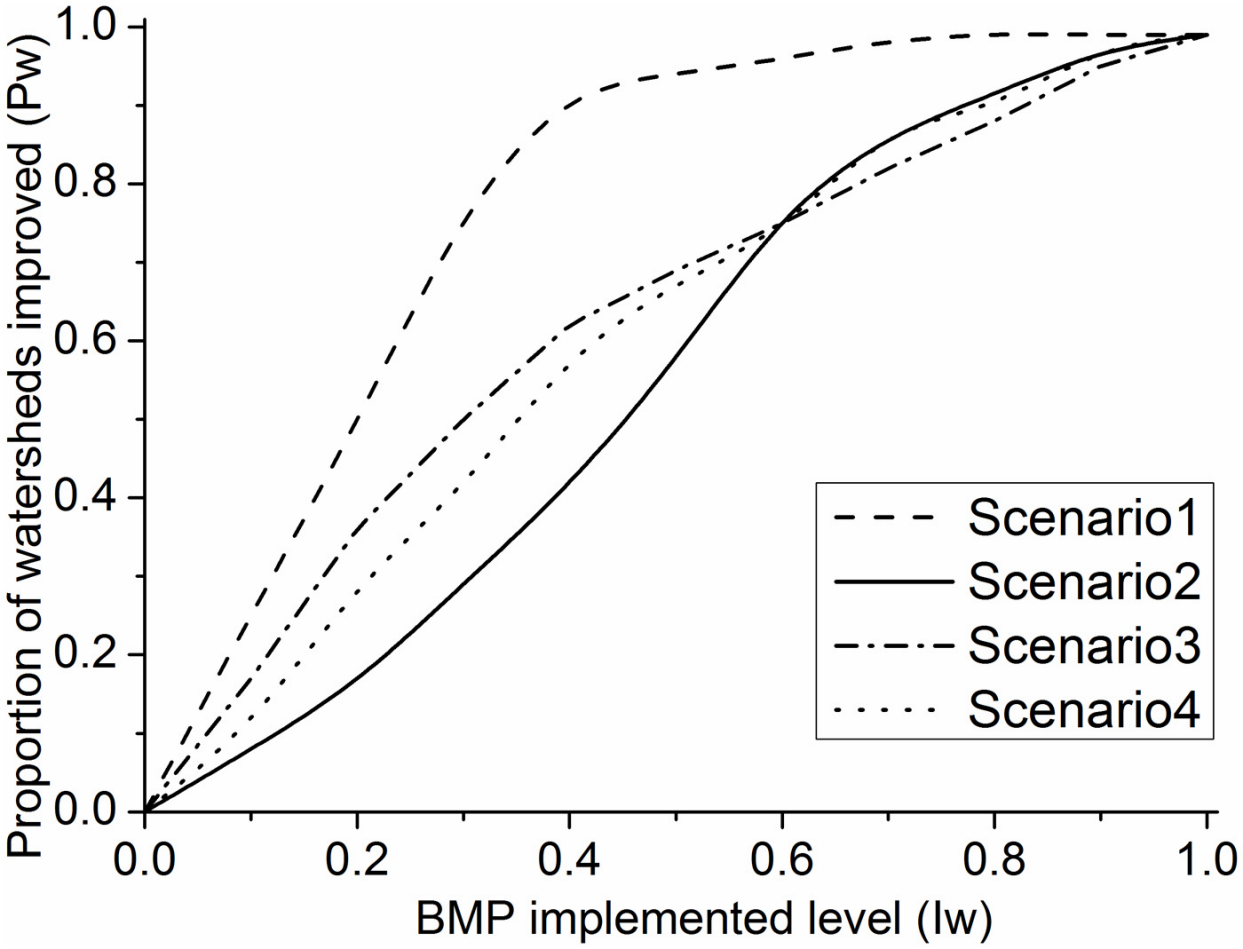
Cost-effectiveness curves for the *P*_*w*_ and *I*_*w*_ under four BMP scenarios.

In terms of the cumulative proportion of subwatersheds where a statistically significant reduction in stream P concentration was observed (*P*_*w*_, second index) (Fig 12) (S6 File), Scenario 1 exhibited the best performance for water quality improvement and provided the most benefit at all levels of BMP implementation (P loss followed a log-normal distribution at the field scale). The performance of Scenario 3 is slightly better than that of Scenario 4 at BMP effort of 0.6 because of the high BMP efficiency of P losses in Scenario 3 (Fig 12) (S6 File); the fields with high P losses exhibit aggregated distributions. Scenario 2 exhibits minimal improvement in water quality when *I*_*w*_ is below 0.6, but its performance is better than that of Scenarios 3 and 4 at the threshold (*I*_*w*_ = 0.6). Therefore, Scenario 1 is the most beneficial BMP allocation for P loss control. P load reduction in the entire watershed can reach 26%, and the probability of obtaining a statistically significant improvement in water quality can reach 51%.

### 4.5 Sensitivity analysis

We conducted three types of sensitivity analysis to confirm the stability and performance of the modeled framework. First, to consider the uncertainty of BMP performance in nutrient control caused by site-specific conditions, BMP reduction (*R*_*f*_) was varied from 40% to 80%. Second, the frequency distributions of P load were modified to normal or lognormal at field and subwatershed scales. Third, the sample number (from 12 to 48) of pre- and post-BMP implementation was adjusted.

Sensitivity analysis shows that Scenario 1 is a robust tool for the spatial allocation of BMPs. Scenario 1 has the highest value of *P*_*w*_ and *R*_*w*_ compared with the other scenarios, and its BMP placement level is 20% (Table 4). By modifying P loss distribution to a normal distribution at the subwatershed scale, the cumulative probability of *P*_*w*_ and *R*_*w*_ decreased in Scenario 1. Similarly, this phenomenon became more obvious when P loss was log-normally distributed. Therefore, all the above results indicate that BMP allocations with an aggregated and targeted structure are useful for P loss control in a mixed-use, landscape-type agricultural watershed.

**Table 4:** Performance of four allocation scenarios according to two benefit indices under different parameter levels.

Scenario parameters				Measured water quality change index				Modeled pollutant reduction index			
*R*_*f*_	*N*	Subwatershed Pdistribution	Field Pdistribution	Scenari1	Scenario 2	Scenario 3	Scenario 4	Scenario 1	Scenario 2	Scenario 3	Scenario 4
0.8	24	Log-normal	Log-normal	0.69	0.20	0.34	0.45	0.43	0.17	0.40	0.30
0.65	48	Log-normal	Log-normal	0.56	0.19	0.32	0.41	0.25	0.15	0.24	0.23
0.65	24	Log-normal	Log-normal	0.51	0.17	0.28	0.39	0.26	0.15	0.36	0.24
0.65	12	Log-normal	Log-normal	0.41	0.16	0.24	0.36	0.26	0.15	0.23	0.20
0.5	24	Log-normal	Log-normal	0.41	0.17	0.24	0.36	0.24	0.13	0.22	0.21
0.4	24	Log-normal	Log-normal	0.20	0.16	0.14	0.18	0.20	0.11	0.18	0.17
0.65	24	Log-normal	Normal	0.38	0.17	0.23	0.39	0.20	0.15	0.26	0.24
0.65	24	Normal	Log-normal	0.43	0.17	0.27	0.39	0.21	0.15	0.28	0.24
0.65	24	Normal	Normal	0.28	0.17	0.23	0.39	0.21	0.15	0.20	0.18

*R*_*f*_: P load reduction after BMP implemented at field scale; *N*: Number of sample before and after BMP implemented; benefit index values (proportions) are reported for a 20% watershed-scale BMP implementation level BMP implementation level.

### 4.6 Visualization of spatial optimal placement of BMPs

**Fig 13:**
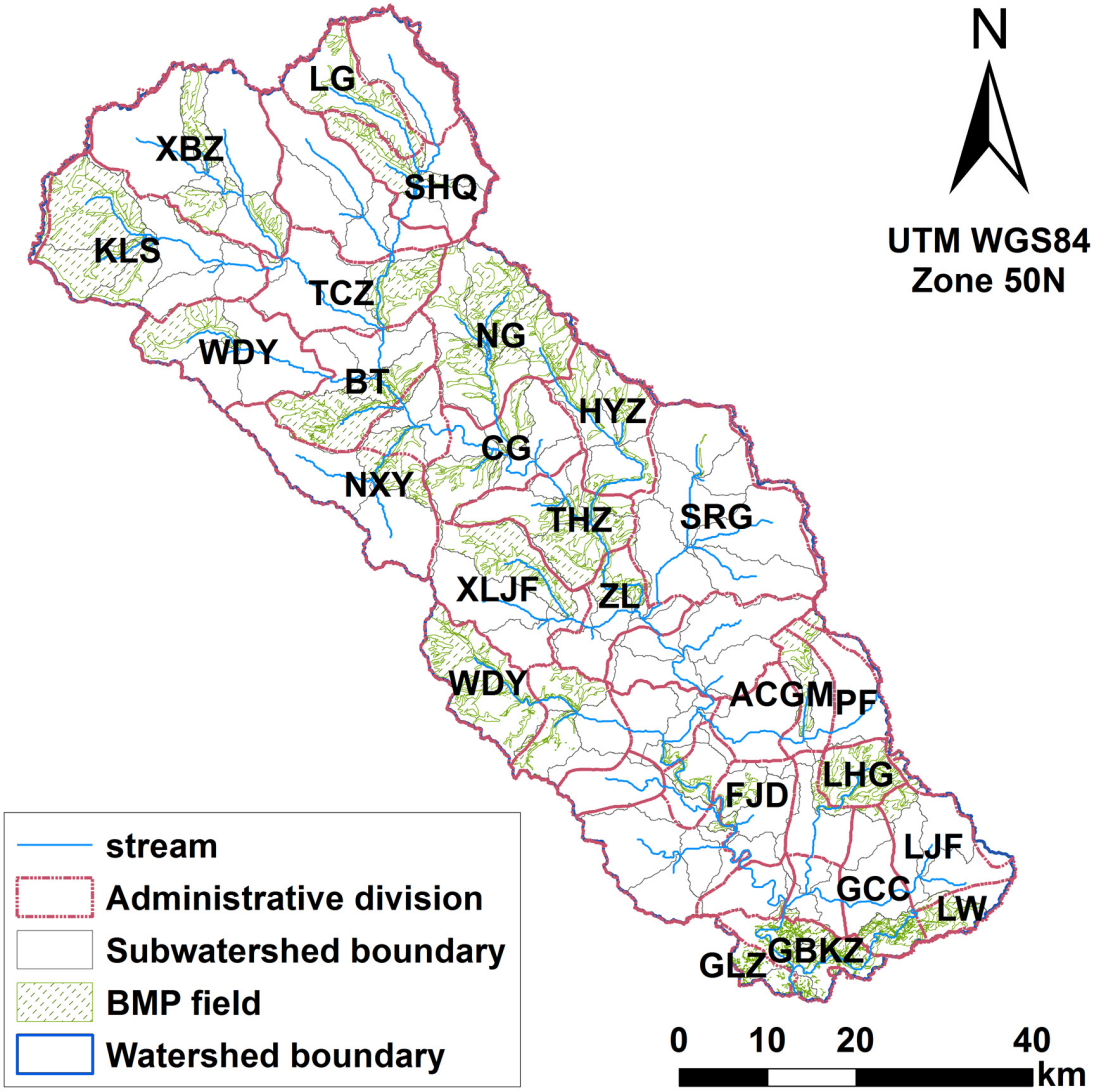
Distributions of BMP implemented based on scenario 1.

The visualization of BMP allocation was processed with Scenario 1 (Fig 13). We also determined the proportion of BMP implementation in each township by overlaying the natural hydrological boundary and township administrative divisions through ArcGIS 10.1 (Table 5). The average watershed area with BMPs was 21%, which is consistent with the assumption of Scenario 1. BMP-implemented areas, namely, Nan Guan (NG), Ku Longshan (KLS), Wu Daoying (WDY), Bai Ta (BT), and Xi Liangjianfang (XLJF), required a much larger proportion of watershed area (49%). These five areas are located in the upper stream of Chaohe River Watershed. Five other townships with over 30% BMP implementation are located in the mid-upper stream of Cheohe River Watershed. Therefore, the critical areas for BMP placement and practical implementation are aggregated and targeted in the mid-upper stream of Chaohe River Watershed.

**Table 5:** Proportion of the BMP implemented at each township.

Township	Abbreviation	Area_township (km^2^)	Area_BMP implemented(km^2^)	Percentage
Anchungoumen	ACGM	60	15	25%
Baita	BT	163	59	36%
Change	CG	154	21	14%
Fujiadian	FJD	86	5	6%
Gubeikou	GBKZ	89	20	22%
Guchengchuan	GCC	104	21	20%
Gaolinzhen	GLZ	27	2	7%
Houyingzi	HYZ	133	29	22%
Kulongshan	KLS	282	93	33%
Leguo	LG	96	23	24%
Lahaigou	LHG	56	16	29%
Liangjianfang	LJF	117	9	8%
Laowa	LW	38	10	26%
Nanguan	NG	227	94	41%
Nanxinying	NXY	181	25	14%
Pingfang	PF	70	5	7%
Shanghuangqi	SHQ	133	46	35%
Shirengou	SRG	340	2	1%
Tuchengzi	TCZ	174	34	20%
Tahuangzhen	THZ	143	44	31%
Wudaoying	WDY	297	78	26%
Xiaobazi	XBZ	304	32	11%
Xiliangjianfang	XLJF	191	47	25%
Zhailing	ZL	100	18	18%
				Mean: 21%

## Discussion

### 5.1 Nonlinear relationship between P load reduction and probability of obtaining a statistically significant P concentration

We employed a sigmoid curve to determine the threshold relationship between *P*_*subw*_ and *R*_*subw*_ at the field scale and the relationship between *R*_*w*_ and *I*_*w*_ and *P*_*w*_ and *I*_*w*_ at the watershed scale. The results indicate that scenarios with a targeted structure significantly reduce P loads and lead to an improvement in water quality at the watershed scale (Fig 12) (Fig 13) (S6 File). Moreover, the relationship between nutrient level and water quality is often nonlinear in Chaohe River Watershed.

For the threshold value, sigmoid curves are usually defined by the mean value (*μ*) and steepness (*S*) of the response and predictor variables. In this study, if 70% to 80% of watershed P load is derived from 1% to 2% of the watershed area, a relatively small P reduction level at the watershed scale could result in a large increase in the probability of water quality improvement [35]. If a high threshold exists, then these watersheds require a relatively high P load reduction to achieve specific P concentrations.

### 5.2 BMP allocation scenarios

Comparison of the four scenarios employed in this study indicates that Scenario 2 does not have any structural components, such as aggregated and targeted, at the field or subwatershed scale. Consequently, it can serve as the baseline scenario to compare the other three scenarios. Obviously, adding a targeted or aggregated structural component to the baseline scenario will improve the effectiveness of BMP placement (Fig 12) (Fig 13) (S6 File). Similarly, BMPs implemented with a targeted structure will produce relatively high P reduction efficiencies with an aggregated structure.

BMP allocations with both targeted and aggregated components were found to be the most cost efficient for improving water quality while ensuring the economic feasibility of solution measures [42]. Aggregated and targeted components can be regarded as a streamlined approximation—selection process of the potential field for BMPs implemented at the field and subwatershed scales; effectiveness is merely based on the specific field that has already been implemented with BMP. Using a different BMP allocation method at field (aggregated) and subwatershed (targeted) scales would allow this method to perform a process similar to the actual iterative process but without the need to re-evaluate site-scale instantaneous efficiency in each step [35]. Furthermore, a layered BMP configuration system for identifying potential sites for BMP implementation can reduce assessment costs.

### 5.3 Practical application of BMP scenarios

The aggregated and targeted components in each scenario are based on models that describe how P load is distributed, how it can be reduced, and what mitigation outcomes might occur [35]. In the actual application, this integrated model system may also be affected by other uncontrollable factors similar to any model-based management approach. First, the influence of weather on BMP effectiveness was not accounted for in this analysis [43]. Second, government policies that result in inconsistent application of regulations or incentives (often unpopular) and the willingness of stakeholders to implement BMPs were also not considered.

## Conclusions and Future Research

A BMP allocation priority framework that involves the use of a BMP tool and a statistical simulation technology informed by HSPF and PI was developed in this study. The proposed approach significantly accelerates the optimization process and thus allows for the testing of a broad-area watershed with hundreds of unique hydrological locations. The approach was tested in Chaohe River Watershed. A practical BMP allocation plan was also developed for P loss mitigation at field and subwatershed scales.

A BMP toolbox was developed to provide site-specific estimates of BMP effectiveness [33]. The estimates focus on site conditions and management practices in China and are based on data from published research. Statistically significant improvement in the water quality of the watershed was considered in BMP effectiveness instead of the water quality of streams.

Two benefit indexes were presented to address the important relationship among water quality, P load reduction, and BMP implementation level as well as to establish a linkage among multiple spatial scales. This condition assisted in the decision-making process by supporting the analysis of the sigmoid curves of these two indexes at field and watershed scales. The threshold of four BMP placement allocations were compared to identify the most cost-effective scenario for BMP implementation. The sensitivity analysis demonstrates that Scenario 1 is stable, robust to uncertainties in model parameters, and efficiently improves water quality. Hence, it is the ideal BMP allocation plan.

The methodology developed in this study can be extended to other watersheds to prioritize BMP allocation for control. However, further research is required. For example, the influence of terrestrial factors and weather on BMP effectiveness in a large watershed should be considered. Moreveor, stakeholders’ interests in BMP placement at the field scale should be represented.

## Supporting Information

S1 File Data source for development of BMPs Tool. (XLSX)

S2 File P load data of Chaohe river watershed (as shown in Fig 5). (XLSX)

S3 File Spatial distribution of PI at field scale from PI model (as shown in Fig 6). (XLSX)

S4 File Test results from Kruskal Wallis method for the probability of detecting a statistically significant difference (P>0.01) in stream phosphorus concentration is plotted against the proportional phosphorus load reduction (as shown in Fig 10). (XLSX)

S5 File Cost—effectiveness curves for the *R*_*w*_ and *I*_*w*_ under four BMP scenarios (as shown in Fig 11). (XLSX)

S6 File Cost-effectiveness curves for the *P*_*w*_ and *I*_*w*_ under four BMP scenarios (as shown in Figs 12 and 13). (XLSX)
